# The prognostic role of tumor size in early breast cancer in the era of molecular biology

**DOI:** 10.1371/journal.pone.0189127

**Published:** 2017-12-06

**Authors:** Anaid Anna Kasangian, Giorgio Gherardi, Elena Biagioli, Valter Torri, Anna Moretti, Elena Bernardin, Andrea Cordovana, Gabriella Farina, Annalisa Bramati, Sheila Piva, Maria Chiara Dazzani, Emanuela Paternò, Nicla Maria La Verde

**Affiliations:** 1 ASST Fatebenefratelli Sacco PO Fatebenefratelli Breast Surgery Unit, Milan, Italy; 2 ASST Fatebenefratelli Sacco PO Fatebenefratelli Department of Pathology, Milan, Italy; 3 IRCCS—Mario Negri Institute for Pharmacological Research, Milan, Laboratory of Methodology for Biomedical Research, Milan, Italy; 4 ASST Fatebenefratelli Sacco PO Fatebenefratelli Department of Oncology, Milan, Italy; University of North Carolina at Chapel Hill School of Medicine, UNITED STATES

## Abstract

**Background:**

The prognosis of early breast cancer (EBC) depends on patient and tumor characteristics. The association between tumor size, the largest diameter in TNM staging, and prognosis is well recognized. According to TNM, tumors classified as T2, could have very different volumes; e.g. a tumor of 2.1 cm has a volume of 4500 mm^3^, while a tumor of 4.9 cm has a volume of 60.000 mm^3^ even belonging to the same class. The aim of the study is to establish if the prognostic role of tumor size, expressed as diameter and volume, has been overshadowed by other factors.

**Methods:**

The primary objective is to evaluate the association between tumor dimensions and overall survival (OS) / disease free survival (DFS), in our institution from January 1^st^ 2005 to September 30^th^ 2013 in a surgical T1-T2 population. Volume was evaluated with the measurement of three half-diameters of the tumor (a, b and c), and calculated using the following formula: *4/3π x a x b x c*.

**Results:**

341 patients with T1-T2 EBC were included. 86.5% were treated with conservative surgery. 85.1% had a Luminal subtype, 9.1% were Triple negative and 7.4% were HER2 positive. Median volume was 942 mm^3^ (range 0.52–31.651.2). 44 patients (12.9%) relapsed and 23 patients died. With a median follow-up of 6.5 years, the univariate analysis for DFS showed an association between age, tumor size, volume, histological grading and molecular subtype. The multivariate analysis confirmed the statistically significant association only for molecular subtype (p 0.005), with a worse prognosis for Triple negative and HER2 positive subtypes compared with Luminal (HR: 2.65; 95%CI: 1.34–5.22). Likewise for OS, an association was shown by the multivariate analysis solely for molecular subtype (HER2 and Triple negative vs. Luminal. HR: 2.83; 95% CI:1.46–5.49; p 0.002).

**Conclusions:**

In our study, the only parameter that strongly influences survival is molecular subtype. These findings encourage clinicians to choose adjuvant treatment not based on dimensional criteria but on biological features.

## Introduction

Breast Cancer (BC) is the most frequent malignancy in the Italian female population, with 50.000 new cases in 2016 [1]. BC has always been staged using the American Joint Committee on Cancer (AJCC) TNM (Tumor Nodes Metastasis) system, developed in 1959 and periodically reviewed till the update to the current seventh edition. The role of T (tumor) dimension has always been well established, and the correlation between T size and patients’ survival was linear and independent from nodal status [2]. Nowadays the prognosis of early breast cancer (EBC) is not related only to the anatomical extension of the disease but it depends on some biological features, concerning intrinsic tumor behaviour such as hormone receptor expression (estrogen and progesterone receptor), HER2 positive expression, ki-67 status and histological grading. In clinical practice, the expression or the absence of these elements allows us to distinguish between different cancer subtypes (Luminal A, Luminal B, HER2 positive and Triple negative breast cancers), which require different therapeutic approaches [3]. The strength of molecular subtyping is well recognised and is constantly evolving: several subgroups are being identified and new therapies are becoming available.

In this new scenario, a deeper reflection on T role is warranted and it is necessary to establish how its prognostic role has evolved in these last years with the use of biological classifications in clinical practice. T is represented by the largest diameter of the invasive cancer component, it has been adopted by the TNM classification because it is easy to obtain and is considered the best way to measure the risk of cancer metastases and recurrence. However, it may not be representative of the real tumor burden; in fact the amount of cancer cells is better described by an approximation of the Tumoral Volume (TV) obtained by the ideal ellipsoid generated from the three spatial dimensions of the invasive tumor measured at the surgical pathology examination [4]. In fact, turning from a one-dimensional to a three-dimensional approach, neoplasms sharing the same greatest length do differ one another due to variation of their height and width.

To assess the appropriate value of the T parameter it’s important to wonder if the currently used largest diameter is effectively the best prognostic model.

## Materials and methods

This is a monoinstitutional, observational study conducted in ASST Fatebenefratelli-Sacco, Presidio Fatebenefratelli, in Milan (Italy). The study was approved by the local Ethical Commettee, “Milano Area B”. The aim of the current study is to explore the prognostic T role in EBC in the era of molecular medicine, using both the largest diameter and TV, to understand if the morphologic parameter is still more relevant than biological features in predicting survival (DFS and OS).

The main inclusion criteria were: presence of histologically confirmed invasive T1-T2 breast cancer and no evidence of axillary node metastatic involvement (clinical and/or ultrasound detection). Exclusion criteria were: male gender; bilateral breast cancer; multicentricity of the tumor; history of previous breast cancer (either invasive or “in situ”); previous neoadjuvant chemotherapy or hormone therapy; inflammatory breast cancer presentation; synchronous metastasis at diagnosis.

We retrospectively analysed data about EBC patients who underwent breast surgery from January 1^st^ 2005 to September 30^th^ 2013. We collected data on primary tumor, including histologic type, histologic grading, ki-67, estrogen receptor (ER) and progesterone receptor (PgR) expression, HER2 gene amplification, peritumoral lymphovascular invasion (LVI). Tumor molecular subtypes were classified as Luminal A and B, Triple negative, Her2 positive following the Maisonneuve classification. Tumor size was calculated both as maximum diameter (according to TNM) in mm and as volume in mm^3^.

The surgical sample was always received fresh for immediate pathological evaluation and sampling. The area containing the tumor was sectioned in sequence at the pathology bench with an interval of about 3 mm and block locations recorded [5]. The entire tumor was blocked out in such a way that the location of each block could be determined. Tissue slices were then fixed and embedded in paraffin as routine. The calculation of the three dimensions (in millimetres) of the invasive component of the tumor was based upon histological examination of haematoxylin-and-eosin stained paraffin sections prepared from the sequential blocks. The maximum length was obtained by summing up the number of histological tissue slices containing the invasive tumor, while the maximum depth and width were measured by light microscopic examination of the sequential histological slides. Once we obtained these three measurements, we assumed that the tumor shape was approximately like a tri-axial ellipsoid with distinct semi axis lengths a > b > c, so the volume of this solid can be calculated using the formula *4/3π x a x b x c*.

### Statistical analysis

Patient and tumor characteristics (age, tumor size, TV, histological grade, subtype, positivity of sentinel nodes and LVI) are expressed as absolute and relative frequencies for categorical variables and as mean, standard deviation (SD), minimum and maximum values for continuous variables.

Cox proportional-hazards regression models were performed to evaluate the influence of patient and tumor characteristics such as TV or tumor size on DFS and OS. First, we used univariate models to identify independent variables that influence prognosis, then we used multivariate models that included variables from the univariate analyses that were related (p<0.10) to DFS or OS. Results were expressed as hazard ratios (HRs) and relative 95% confidence intervals (CIs). Analyses were performed with SAS statistical software (version 9.2). The DFS and OS were described with the Kaplan-Meier method.

## Results

A total of 341 patients were included in the analysis, with a median age of 60.6 years old (29.9–87.7), 77.1% of them were postmenopausal. 86.5% had been treated with conservative breast surgery (quadrantectomy or lumpectomy), followed by adjuvant radiotherapy, while 13.5% underwent mastectomy. The median number of sentinel lymph nodes (SLN) excised was 1.6 (range from 1 to 7) and in 218 cases (63.9%) only one single SLN was excised. 25.2% of patients had at least one positive SLN and a total of 61 axillary dissections were performed.

The most frequent histologic type was ductal carcinoma (59.2%). The median measure of the largest tumor diameter was 15 mm; median TV was 942 mm^3^ (range 0.52–31651.2 mm^3^). The most frequent molecular subtype was Luminal (83.4%), followed by Triple negative (9.1%) and HER2 positive (7.4%). In most cases an intermediate-advanced histological grade was reported (80.7%). LVI was documented in 111 specimens (32.6%). Tables 1 and 2 describe the characteristics of patients, tumors and surgery.

**Table 1:** Characteristics of patients and surgery.

**Patients–N (%)**	341 (100.0)
**Age at surgery (years)**	
Mean (SD)	60.6 (11.5)
Min-max values	29.9–87.7
**Menopausal Status–**N (%)	* *
Pre	78 (22.9)
Post	262 (77.1)
**Type of surgery–N (%) Mastectomy**	46 (13.5)
Mastectomy	46 (13.5)
Quadrantectomy	286 (83.9)
Nodulectomy	9 (2.6)
**Number of excised sentinel nodes**	
Mean (SD)	1.6 (0.9)
Min-max values	01-lug
**Sentinel nodes excised–N (%)**	
1	218 (63.9)
2	76 (22.3)
≥5	45 (13.2)
**Number of positive sentinel nodes**	
Mean (DS)	1.2 (0.5)
Min-max values	01-apr
**Sentinel node status–N (%)**	* *
Negative sentinel node	255 (74.8)
*Isolated tumor cells*	*33 (12*.*9)*
*No Isolated tumor cells*	*222 (87*.*1)*
Positive sentinel node	86 (25.2)
*Micrometastases*	*30 (35*.*7)*
*Macrometastases*	*54 (64*.*3)*
Axillary dissection–N (%)*	61 (17.9)
Number of resected nodes	
*Mean (DS)*	*15*.*4 (5*.*6)*
*Min-max values*	*ago-31*
Number of positive nodes	
*Mean (DS)*	*1*.*9 (3*.*6)*
*Min-max values*	*0–18*

**Table 2:** Tumor characteristics.

**Histology–N (%)**	
Lobular	54 (15.8)
Mixed	49 (14.4)
Ductal	202 (59.2)
Other*	36 (10.6)
**Tumor size (in mm)**	
Median	15.0
Min-max	1.0–43.0
**TV (in mm**^**3**^**)**	
Median	942.0
Min-max	0.52–31651.20
**ki 67**	
Mean (SD)	16.14 (11.8)
Min-max values	5.00–80.00
**ki 67 –N (%)**	
Low (0%-13%)	188 (55.1)
Intermediate (14%-19%)	57 (16.7)
High (≥20%)	96 (28.2)
**ER (%)**	
Mean (DS)	63.4 (34.5)
Min-max values	0.00–100.00
**ER positive–N (%)**	284 (83.3)
**PgR (%)**	
Mean (SD)	48.8 (37.2)
Min-max values	0.00–100.00
**PgR positive–N (%)**	261 (76.5)
PgR ≥20%	227 (66.6)
**HER2 positive**–**N (%)**	49 (14.4)
**Histological grade–N (%)**	
1	66 (19.4)
2	168 (49.3)
3	107 (31.4)
**Subtype–N (%)**	
Luminal	281 (83.4)
*Luminal A*	*176 (52*.*2)*
*Luminal B*	*105 (31*.*2)*
HER2 positive	25 (7.4)
Triple negative	31 (9.1)
Undetermined	4
**Presence of LVI–N (%)**	111 (32.6)

TV, Tumor Volume; ER, Estrogen Receptor; PgR, Progesterone Receptor; LVI, Lymph Vascular Invasion

*description (mucinous; tubular; apocrine; medullary; papillary)

Table 3 shows the distribution of molecular subtypes in the subgroup of patients that had undergone a mastectomy. A Cox regression analysis to evaluate the influence of type of surgery (mastectomy vs other surgeries) on DFS was performed but no remarkable results were observed (HR: 0.86[95%CI: 0.34–2.18] p = 0.757).

**Table 3:** Type of surgery according to molecular subtype.

Subtype- N%	Mastectomy (N = 46)	Quadrantectomy (N = 295)
**Luminal A**	14 (31.1)	162 (54.9)
**Luminal B**	18 (40.0)	87 (24.5)
**HER2 positive**	6 (13.3)	19 (6.4)
**Triple negative**	7 (15.6)	24 (8.1)
**undetermined**	1	3

In Table 4 we describe adjuvant treatment, chosen according to clinical practice. Radiotherapy was performed in 276 patients (80.9%).

**Table 4:** Adjuvant therapy administered.

Treatment	N (%)
**Only hormonal therapy–**N (%)	216 (63.3)
**Only chemotherapy–**N (%)	56 (16.4)
**Hormonal therapy and chemotherapy–**N (%)	60 (17.6)
**None (except for radiotherapy)**–N (%)	9 (2.6)

**Fig 1:**
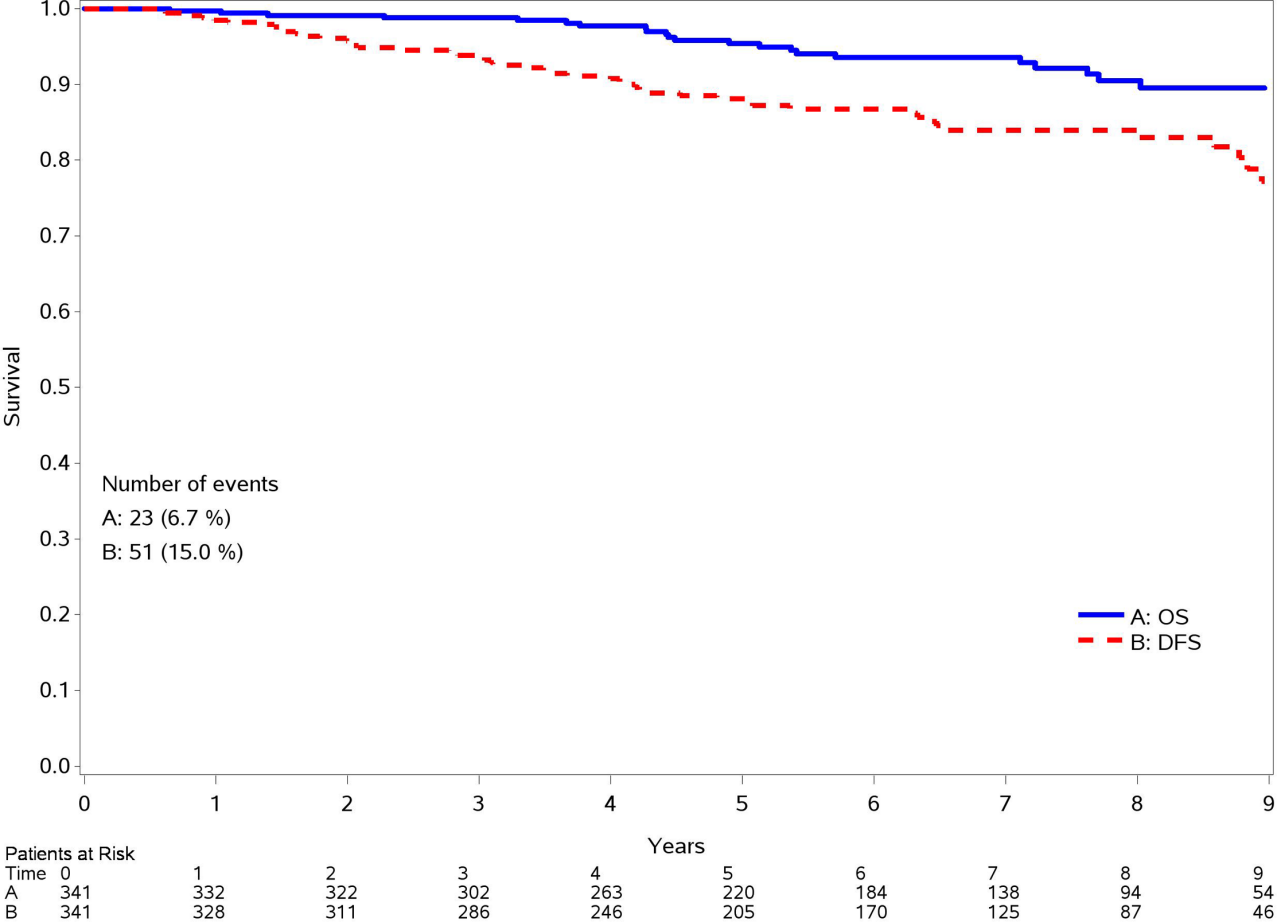
OS and DFS.

Median follow-up was of 6.5 years (range 4.5–8.4). In this period 44 patients relapsed, so 87.1% of patients are alive without a local or distant recurrence 93.8% are alive while 23 patients died. (Fig 1)

A univariate analysis was performed to evaluate the association between prognostic factors and OS/DFS. (Tables 5 and 6). Age seems to be inversely associated with DFS (HR: 0.74; 95%CI: 0.58–0.95; p 0.016), while major diameter or higher volume have a statistically significant negative impact on DFS (HR: 1.45; 95%CI: 1.03–2.05; p 0.032; and HR: 1.64; 95%CI: 0.95–2.84; p 0.078). Also grading (2 and 3 vs. grade 1 HR: 2.65; 95%CI: 1.53–4.59; p 0.001) and molecular subtype (Her2+ and Triple negative vs Luminal tumors HR: 3.79; 95%CI: 2.16–6.66; p <0.001) seems to influence DFS, but at the multivariable analysis only molecular subtype seems to independently influence the DFS with a HR of 2.63 (95%CI: 1.33–5.18; p 0.005) or a HR of 2.65 (95%CI: 1.34–5.22; p 0.005), respectively for the model with tumor size or TV, corrected for the other prognostic factors. In fact, patients with Triple negative BC and HER2 positive tumors have a significantly worse prognosis than patients with a Luminal subtype in both the multivariable models used.

**Table 5:** Association with DFS (Cox proportional hazard model).

	Univariate		Multivariate			Multivariate		
	HR (95%CI)	P Value	HR (95%CI)		P value	HR (95%CI)		P value
**Age** (increase of 10 years)	0.74 (0.58*–*0.95)	**0.016**	0.83 (0.64–1.07)		0.148	0.83 (0.64–1.07)		0.144
**Tumor size** (increase of 1 cm)	1.45 (1.03–2.05)	**0.032**	1.08 (0.74–1.58)		0.683			
**TV** (increase of 10 cm^3^)	1.64 (0.95–2.84)	**0.078**				1.07 (0.56–2.05)		0.837
**Histological grade**								
1 +2 (reference)	1							
3	2.65 (1.53–4.59)	**0.001**	1.54 (0.78–3.03)		0.215	1.56 (0.80–3.04)		0.190
**Subtype**								
Luminal	1							
HER2 + and Triple negative	3.79 (2.16–6.66)	**<0.001**	2.63 (1.33–5.18)		**0.005**	2.65 (1.34–5.22)		**0.005**
**Positive SLN**								
No metastases	1							
Metastases	1.58 (0.89–2.81)	0.117						
**Presence of LVI (yes vs. no)**	1.55 (0.88–2.75)	0.131						

TV, Tumor Volume; LVI, Lymph Vascular Invasion; SLN Sentinel Lymph Node

**Table 6:** Association with OS (Cox proportional hazard model).

	Univariate		Multivariate	
	HR (95%CI)	p-value	HR (95%CI)	p-value
**Age** (increase of 10 years)	0.81 (0.56–1.16)	0.241		
**Tumor size** (increase of 1 cm)	1.19 (0.68–2.08)	0.544		
**TV** (increase of 10 cm^3^)	1.40 (1.51–3.87)	0.515		
**Histological grade**			1.70 (0.89–3.24)	0.108
1 and 2	1			
3	2.70 (1.18–6.16)	**0.018**		
**Subtype**				
Luminal	1			
HER2 + and Triple negative	16.45 (2.82–14.74)	**<0.001**	2.83 (1.46–5.49)	**0.002**
**SLN positivity**				
No metastases	**1**			
Metastases	1.54 (0.65–3.63)	0.324		
**Presence of LVI (yes vs. no)**	1.57 (0.66–3.72)	0.307		

TV, Tumor Volume; LVI, Lymph Vascular Invasion

**Fig 2:**
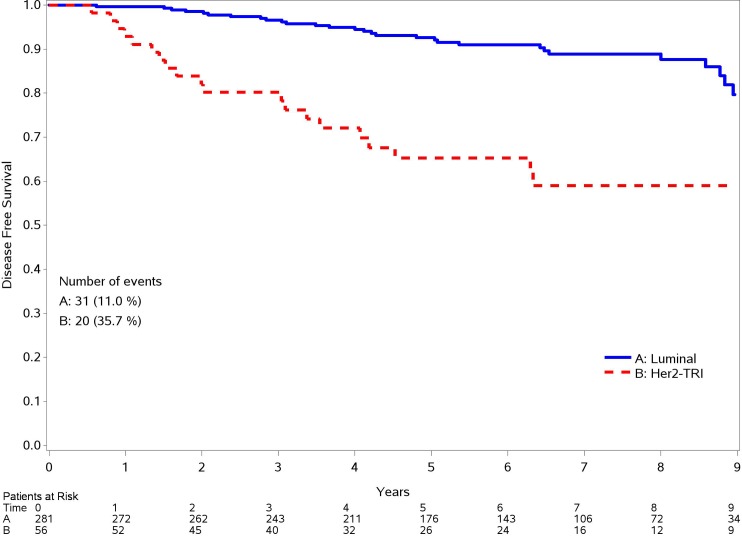
DFS for different molecular subtype.

**Fig 3:**
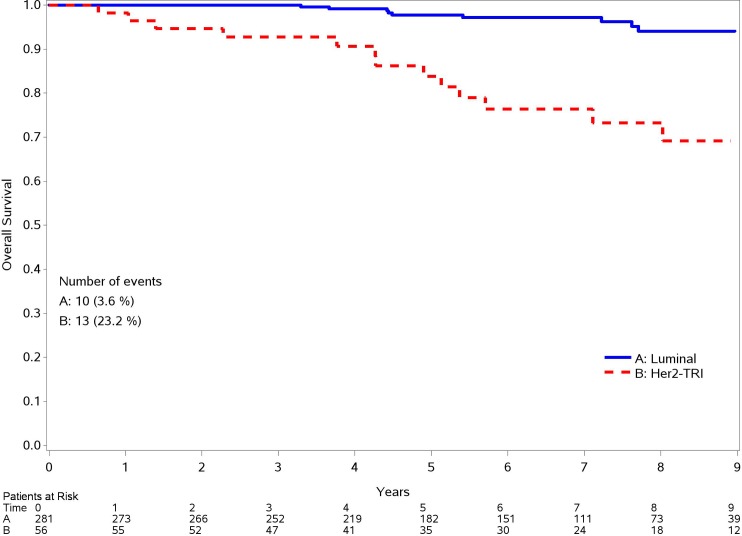
OS for different molecular subtype.

Regarding OS, a significant association is shown for grading (2 and 3 vs. grade 1 HR: 2.70; 95% CI: 1.18–6.16; p 0.018) and molecular subtype (HER2 positive and Triple negative vs Luminal tumors HR: 16.45; 95%CI: 2.82–14.74; p 0.001) at the univariate analysis, but considering the multivariate one, only molecular subtype remains associate with OS (HR: 2.83; 95%CI:1.46–5.49; p 0.002), as it happens for DFS. In Figs 2 and 3 OS and DFS curves by molecular subtype are shown.

In order to better understand the Luminal breast cancer subgroup, which is the largest part of our series, we described the distribution of tumor size in the Luminal A and Luminal B subgroups (N = 176 and N = 105 respectively) in Table 7. Tumor size seems to impact on DFS when the analysis is performed in the Luminal A subgroup. The association is confirmed also in the multivariate model with SLN status, as shown in Table 8. Instead in Luminal B tumors, the only factor associated with DFS is age (Table 9).

**Table 7:** Distribution according to tumor size in Luminal A and Luminal B tumors.

Tumor size—N (%)	Luminal A	Luminal B
**>1–5 mm**	9 (5.1)	2 (1.9)
**>5–10 mm**	40 (22.7)	19 (18.1)
**>10–20 mm**	99 (56.3)	59 (56.2)
**>20 mm**	28 (15.9)	25 (23.8)

**Table 8:** Association with DFS in the Luminal A patients (Cox proportional hazard model).

	Univariate		Multivariate	
	HR (95%CI)	P Value	HR (95%CI)	P Value
**Age** (increase of 10 years)	1.47 (0.89–2.42)	0.131		
**Tumor size (increase of 1 cm)**	2.24 (1.31–3.85)	**0.003**	1.91 (1.09–3.51)	**0.024**
**Volume** (increase of 10 cm3)	2.45 (0.99–6.08)	0.053		
**Histological grade (3 vs 2+1)**	3.00 (0.93–9.63)	0.066		
**Positive SLN (metast vs no metast)**	3.87 (1.40–10.68)	**0.009**	2.88 (1.00–8.30)	0.051
**Presence of LVI (yes vs no)**	2.26 (0.78–6.55)	0.134		

**Table 9:** Association with DFS in the Luminal B patients (Cox proportional hazard model).

	Univariate	
	HR (95%CI)	P Value
**Age** (increase of 10 years)	0.57 (0.36–0.89)	**0.013**
**Tumor size (increase of 1 cm)**	0.95 (0.43–2.12)	0.907
**Volume** (increase of 10 cm3)	1.51 (0.39–5.95)	0.555
**Histological grade (3 vs 2+1)**	0.85 (0.28–2.55)	0.775
**Positive SLN (metastic vs no metastatic)**	1.39 (0.47–4.16)	0.552
**Presence of LVI (yes vs no)**	0.50 (0.14–1.82)	0.296

## Discussion

The role of TV has been studied either as a staging parameter to measure the dimension of the tumor properly, or as a parameter to monitor the efficacy of therapy in different solid tumors. Boggs et al, in esophageal cancer, stated that volume was a significant multivariate predictor for improved local disease control, DFS and OS; besides it is a more powerful predictor of patient outcome than traditional TNM staging. [6] In patients with advanced nasopharyngeal carcinoma the measurement of the primary TV has been reported as an important prognostic factor [7]. Moreover, TV could have very different values within the same T-stage classification [8], due to the different and irregular shape that the tumor bulk can have in many types of cancer (e.g., malignant pleural mesothelioma and recurrent malignant glioma) in which tumor size is difficult to express using only tumor diameter [9–10]. This problem could be overcome by using simple devices, such as manual delineation of target areas on 3D cross-sectional images, especially for cancers studied with a CT scan or MRI in patients who undergo radiation therapy, for which the “gross tumor volume” is contoured by the Radiation Oncologist [6].

In EBC, the tumor size has a well-known prognostic role and can be evaluated as cTNM for patients who will undergo neoadjuvant treatment or as pTNM for patients who receive upfront surgery. Veronesi et al explore this concept, highlighting some issues aimed at improving the TNM staging system. In particular, T parameter may be the same for tumors with very different prognosis within the same category: a patient with a T2 tumor of 2.1 cm could have a different prognosis from a patient with a 4.9 cm tumor. The evaluation of TV could fill this gap. For example, Veronesi et al, assuming that breast cancer nodules are spherical calculated approximately that a 2.1 cm T corresponds to a volume of 4500 mm^3^; while a T of 4.9 cm corresponds to a volume of 60000 mm^3^ [11]. In our opinion, only few nodules have a perfect spherical shape. Therefore, neoplastic nodules are three-dimensional solids, correctly determined by the volume of an ellipsoid *4/3π x a x b x c* where a, b and c are the three half-diameters of the tumor. If TV is calculated based only on the major diameter, as it happens in a sphere, it will be overestimated, because the other minor diameters are not considered.

Wapnir et al. reported a population of 165 small breast cancers, less than 2.5 cm and compared the values of TV, calculated as a sphere or as an ellipsoid, in order to demonstrate that the maximum diameter is inadequate because it leads to a wrong approximation of the real tumor burden. [4].

The analysis that we performed in this study, failed to demonstrate that TV has a prognostic role in EBC and doesn’t add any additional information to T, although it could theoretically be a more accurate measurement to estimate the total amount of cancer cells. Moon et al conducted a study on 2250 patients; even they couldn’t prove the prognostic role of TV, but interestingly, they found an influence of genomic on tumor shape. They stated that transcriptome analysis data suggest a potential link between the spatial growth patterns and some specific extra-cellular matrix gene [12]. Besides, the molecular subtypes of breast cancer affect the patterns of the 3-dimensional tumor growth in breast tumors. For example, Triple negative tumors tend to have a round shape and smooth margins when compared to other subtypes [13].

The results of our study showed that age, major diameter, TV, grading and molecular subtype influence DFS in univariate analysis. In multivariate analysis, only molecular subtype showed an association. In the same way, OS was influenced only by molecular subtype. Regarding specifically the Luminal A population, we interestingly observed that the tumor size correlates with prognosis. So only for this subgroup of patients the T parameter maintains its important prognostic role. Furthermore, we have shown that even maximum diameter, which is the first parameter used in the most widespread staging system (TNM -AJCC staging system) doesn’t influence the prognosis in a series of small tumors (T1 and T2). Molecular subtype seems to be the strongest factor, the only statistically significant variable. A possible explanation is that we considered only small size tumors because T3 were excluded from our study and among these small tumors the biology was predominant over tumor dimension. In our cohort, the median size of the tumors was 15 mm ranging between 1 and 43 mm, with a volume range of 0.52 versus 31651.20 mm^3^. So even in our work the molecular profile of the tumor is confirmed to be the most important prognostic factor for both DFS and OS. Consequently, the choice of adjuvant treatment should depend on molecular subtype, more than on tumor diameter, in particular considering specific treatments for HER2 positive and Triple negative BC, which have a worse prognosis.

More and more data in literature support the need for aggressive treatment, such as adjuvant chemo plus antiHER2 treatment in very small tumors, as pT1a, with great benefit [14]. In our series, 94% of patients are alive after 5 years and 87% patients have no disease relapse. This good prognosis may depend on the use of adjuvant treatment (both radiotherapy, hormone therapy and chemotherapy). In particular we observed that many patients with a Luminal molecular profile (22.4% of the population) underwent adjuvant chemotherapy, which could explain this result.

Molecular subtyping becomes both a prognostic and a predictive factor and that’s why this variable is so strong in our results. These findings encourage clinicians to choose adjuvant treatment not based on dimensional criteria but on biological features.
